# Correction: Xu et al. Whole-Genome Sequencing and Genome Annotation of Pathogenic *Elsinoë batatas* Causing Stem and Foliage Scab Disease in Sweet Potato. *J. Fungi* 2024, *10*, 882

**DOI:** 10.3390/jof11030166

**Published:** 2025-02-20

**Authors:** Yuan Xu, Yuqing Liu, Yihan Wang, Yi Liu, Guopeng Zhu

**Affiliations:** 1 Sanya Nanfan Research Institute, Hainan University, Sanya 572025, China; 22210902000008@hainanu.edu.cn (Y.X.); 20213007007@hainanu.edu.cn (Y.L.); 23210902000002@hainanu.edu.cn (Y.W.); 2 Key Laboratory for Quality Regulation of Tropical Horticultural Crops of Hainan Province, School of Tropical Agriculture and Forestry, Hainan University, Haikou 570228, China

In the original article [1], in Section 2.4 of Materials and Methods, “Haidai 7798” was used for the pathogenicity assay; however, the “Fucaishu 1830-11” was used for the pathogenicity assay instead of “Haidai 7798” in our actual experiment. We made a mistake in the process of writing. The corrected paragraph is as follows:

The “Fucaishu 1830-11” seedlings were planted in sterilized nutrient soil and grown in a plant growth incubator (28 °C, 8 h light/16 h dark). The conidial suspension was evenly sprayed onto young leaves, shoots, and stems using a sprayer. Five plants were inoculated, ensuring that each plant was sprayed until the liquid dripped off. Plants inoculated with sterile water served as controls. The inoculated plants were placed in a plastic greenhouse outdoors, where the temperature during the inoculation period ranged from 20 °C to 28 °C, and water was regularly sprayed to maintain a relative humidity of ≥75%. Disease symptoms in the sweet potato plants were observed and recorded daily. The experiment was repeated three times. 

The authors state that the scientific conclusions are unaffected. This correction was approved by the Academic Editor. The original publication has also been updated.
